# Circadian dysfunction in response to *in vivo* treatment with the mitochondrial toxin 3-nitropropionic acid

**DOI:** 10.1042/AN20130042

**Published:** 2014-01-13

**Authors:** Takashi Kudo, Dawn H. Loh, Yu Tahara, Danny Truong, Elizabeth Hernández-Echeagaray, Christopher S. Colwell

**Affiliations:** *Laboratory of Circadian and Sleep Medicine, Department of Psychiatry and Biobehavioral Sciences, University of California, Los Angeles, CA, U.S.A.; †Department of Physiology and Pharmacology, School of Advanced Science and Engineering, Waseda University, Tokyo 162–8480, Japan; ‡Neurofisiología del desarrollo y la neurodegeneración, Unidad de Biomedicina, Universidad Nacional Autónoma de México

**Keywords:** circadian, clock, suprachiasmatic, ACSF, artificial CSF (cerebrospinal fluid), CT, circadian time, DD, constant dark, DIC, differential interference contrast, HD, Huntingon’s disease, i.p., intraperitoneally, LD, light-dark, 3-NP, 3-nitropropionic acid, PD, Parkinson’s disease, SCN, suprachiasmatic nucleus, SFR, spontaneous firing rate, ZT, Zeitgeber time

## Abstract

Sleep disorders are common in neurodegenerative diseases including Huntington's disease (HD) and develop early in the disease process. Mitochondrial alterations are believed to play a critical role in the pathophysiology of neurodegenerative diseases. In the present study, we evaluated the circadian system of mice after inhibiting mitochondrial complex II of the respiratory chain with the toxin 3-nitropropionic acid (3-NP). We found that a subset of mice treated with low doses of 3-NP exhibited severe circadian deficit in behavior. The temporal patterning of sleep behavior is also disrupted in some mice with evidence of difficulty in the initiation of sleep behavior. Using the open field test during the normal sleep phase, we found that the 3-NP-treated mice were hyperactive. The molecular clockwork responsible for the generation of circadian rhythms as measured by PER2::LUCIFERASE was disrupted in a subset of mice. Within the SCN, the 3-NP treatment resulted in a reduction in daytime firing rate in the subset of mice which had a behavioral deficit. Anatomically, we confirmed that all of the treated mice showed evidence for cell loss within the striatum but we did not see evidence for gross SCN pathology. Together, the data demonstrates that chronic treatment with low doses of the mitochondrial toxin 3-NP produced circadian deficits in a subset of treated mice. This work does raise the possibility that the neural damage produced by mitochondrial dysfunction can contribute to the sleep/circadian dysfunction seen so commonly in neurodegenerative diseases.

## INTRODUCTION

It is increasing evident that in neurodegenerative disorders, sleep disruptions are common and occur early in disease progression (Schlosser et al., 2012; Morton, 2013; Willison et al., 2013). In some cases, sleep disturbance will occur years before the onset of dementia or motor symptoms characteristic of the specific disease in question (Iranzo et al., 2006; Julien et al., 2007). While a number of possible mechanisms may underlie these sleep disturbances, dysfunction of the circadian system may be an integral contributing factor. The circadian system generates daily rhythms in behavior and biological processes found throughout the body. At a molecular level, circadian rhythms are generated by robust rhythms in the transcription/translation of clock genes such as *Period 2* (*Per2*) in an auto-regulated series of negative-feedback loops that make up the molecular circadian clock (Maywood et al. 2007; Takahashi et al. 2008a). At a systems level, the central pacemaker is the suprachiasmatic nucleus (SCN) of the hypothalamus. The SCN integrates environmental information (especially blue light) and uses this information to synchronize molecular oscillations found throughout the body. The SCN network generates robust rhythms in spontaneous neural activity which is a critical signal by which the central clock communicates with the rest of the body (Welsh et al. 2010; Colwell, 2011). Disruption of the SCN would impact the temporal patterning of behaviors and physiological systems. For example, the common clinical complaint of Huntington's disease (HD) and Parkinson's disease (PD) patients of difficulty in sleeping at night and staying awake during the day is consistent with a disrupted circadian system. Indeed, evidence for circadian dysfunction has been found in a variety of genetic models of neurodegenerative disorders (Morton et al., 2005; Gonzales and Yin, 2010, Bedrosian et al., 2011; Kudo et al 2011a, 2011b; Oakeshott et al., 2011; Roh et al., 2012; Kantor et al., 2013; Fisher et al., 2013; Loh et al., 2013).

Deficits in mitochondrial function are commonly thought to play a role in the pathogenesis of neurodegenerative disorders (Beal, 2005; Cookson, 2012; Nakamura et al., 2012; Youle and van der Bliek, 2012; Subramaniam and Chesselet, 2013). For example, decreased glucose metabolism, which is suggestive of mitochondrial dysfunction, has been found in symptomatic HD patients (Jenkins et al., 1993; Antonini et al., 1996; Gu et al., 1996; Feigin et al., 2001; Shirendeb et al., 2011). There is compelling evidence that mutant Huntingtin alters mitochondrial trafficking and function (Chang et al., 2006; Cui et al., 2006; Rockabrand et al., 2007; Orr et al., 2008; Shirendeb et al., 2011; Song et al., 2011). Complex II of the mitochondrial electron transport chain appears to be particularly vulnerable in HD (Benchoua et al., 2006) and steps, which are taken to improve mitochondrial function or increase energy metabolism in animal models of HD, have proven promising in reducing the pathology caused by the genetic mutation (Chiang et al., 2012; Damiano et al 2013). The mitochondrial complex II toxin 3-nitropropionic acid (3-NP) blocks succinic dehydrogenase in complex II and produces pathological changes in striatal tissue. This toxin has been widely used to unravel the degenerative process underlying HD because its damage replicates the neuropathological hallmarks seen in this disease (Beal et al., 1993; Brouillet et al., 1999). Thus, 3-NP is a pharmacological tool that can reproduce the phenotype of disorders that exhibit striatal degeneration due to a mitochondrial dysfunction, such as HD, type I glutaric aciduria, and certain inherited metabolic disorders (Brouillet et al., 2005). Of course, a deficiency in energy metabolism may alter function outside of the striatum and recent work suggests that the spontaneous electrical activity of SCN neurons is very sensitive to changes in the redox state (Wang et al., 2012). This finding raises the possibility that SCN neural activity and circadian behavior could be especially sensitive to changes in mitochondrial function.

To examine the impact of the neuropathological changes caused by 3-NP treatment on circadian rhythms, we treated C57BL/6 mice with low doses (15 mg/kg; intraperitoneal injection) of the mitochondrial toxin once a day for 5 days. After allowing the mice to recover, we examined wheel running activity during both light-dark (LD) and constant dark (DD) conditions. The temporal patterning of sleep behavior was monitored using a video analysis system. The locomotor activity of treated mice was also measured in an open field behavioral test during their normal sleep time. Next, we determined the impact of 3-NP treatment on the amplitude and phase of circadian rhythms in *Period2*-driven bioluminescence measured in the SCN, hippocampus, heart, and liver *in vitro*. Furthermore, we examined the spontaneous electric activity of SCN neurons in brain slices prepared from 3-NP-injected mice that had been behaviorally characterized. Finally, we sought to confirm that the 3-NP treatment caused tissue damage in the striatum.

## MATERIALS AND METHODS

### Animals

The experimental protocols used in this study were approved by the University of California, Los Angeles (UCLA) Animal Research Committee and all recommendations for animal use and welfare, as dictated in the UCLA Division of Laboratory Animals and the guidelines from the National Institutes of Health, were followed. We obtained C57BL/6 mice from breeding colonies at UCLA. Animals were placed in chambers where lights were on at 08:00 and off at 20:00 (temperature, 22±2°C). Food and water were supplied *ad libitum*. These mice were placed in this LD cycle upon weaning at 14–21 days of age.

2–4-month-old PER2::LUC knock-in mice on the C57BL/6J background [backcrossed for a minimum of 12 generations (Yoo et al. 2004)] from our breeding colony were used for all experiments. For real-time monitoring of bioluminescence, adult PER2::LUC knock-in male mice were entrained to a 12:12 LD cycle before 3-NP injections.

### Drug injection

Complex II inhibitor, 3-NP (Sigma–Aldrich) was dissolved in phosphate buffer (pH 7.4) and intraperitoneally (i.p., 15 mg/kg) injected into mice. Control mice were administered with saline at a dose of 10 ml/kg. The mice were injected for 5 consecutive days, with injections at Zeitgeber time (ZT) 9 (9 h after lights on). ZT 9 was selected out of convenience and to keep our work consistent with previous studies (Rodríguez et al., 2010). We did not examine other phases of drug administration.

### Wheel running behavior

Male mice (from 8 weeks of age) were housed individually in cages with wheels (23 cm diameter, Mini Mitter), and their wheel-running activity was recorded as revolutions per 3 min interval. The control group (*n*=10) and 3-NP group (*n*=10) were exposed to a 12:12 LD (light intensity, 300 lux at the cage level) for 10 days. Then the animals were placed into DD for 10–20 days to assess their free-running activity pattern. Some mice in DD were exposed to a brief light treatment (white light, 100 lux at the cage level, 10 min) at circadian time (CT) 16, where CT 12 was defined by the locomotor activity onset. After light exposure, the animals were allowed to free-run undisturbed in DD for 10 days. Phase shifts in the activity rhythm were determined by measuring the phase difference between eye-fitted lines connecting the onset of activity for 10 days before and 10 days after an experimental manipulation. Measurements were made by an investigator ‘blind’ to the experimental group. Stimulus intensity (lux) was measured with a light meter (BK Precision). All handling of animals was performed in either the light portion of the LD cycle or DD with the aid of night-vision goggles (FJW Optical Systems).

The locomotor activity rhythm of mice was analyzed by periodogram analysis combined with χ^2^ test with *P*=0.05 significance level (El Temps made by Dr Antoni Diez-Noguera) on the raw data. The periodogram shows the amplitude (=power) of periodicities in the time series for all periods of interest (between 20 and 31 h in 3 min steps). The power values were normalized to the percentage of variance derived from the Qp values of the periodogram (Qp×100/N, where N is the total number of data points) according to the calculated *P*=0.05 significance level. During DD, slopes of an eye-fitted line through the onsets were also used to confirm period estimates made with the periodogram analysis. The duration of each cycle devoted to wheel-running activity is designated α, whereas the duration of no-wheel-running activity is designated ρ. To measure these parameters, the average pattern of activity (i.e. the form estimate) was determined at modulo-period for each animal in DD for 10 days. Then for each wave form, α was calculated as the time during which the motor activity was above the mean. Fragmentation in the form of bouts/day was determined using Clock Lab (Actimetrics), using a setting of max gap at 21 min and threshold at 3 counts per min. With the periodogram analysis, when the power (% variation) was below 35%, we judged the mouse to exhibit a disrupted rhythm.

### Open-field test

Spontaneous activity was measured in both 3-NP-treated and vehicle control groups using the open field test (Crawley, 2000). Each mouse was placed in the center of the cage and the open-field activity was measured for 30 min in a Plexiglas cage (height: 30 cm; length: 25 cm; width: 25 cm) equipped with infrared sensors connected to Versadata software (Accuscan Instruments) that recorded simultaneously horizontal and vertical ambulation, stereotyped movements and total time in movement. Other parameters including immobility time, time spent in the center, and time spent in the periphery were also evaluated at 5 min intervals and results were calculated as the average of the six intervals for a total of 30 min of observation. These experiments were conducted from ZT 3–6 when mice would normally be sleeping.

### Brain slice preparation for electrophysiology

After 3-NP or vehicle treatment, wheel running activity of mice was evaluated as described above and the power of the rhythm noted. Animals were sacrificed between ZT 2 and 3 in the LD cycle for recording during the day. Five mice (30 cells) were examined in the control group, 6 mice (40 cells) in the 3-NP normal (power >35%) group, and 3 mice (12 cells) in the 3-NP disrupted (power < 35%) group. In all cases, mice were sacrificed by deep anesthesia with isoflurane (Clipper Distributing) and rapidly decapitated 1.5 h before recording. To prepare SCN cultures, the brain was quickly excised from the skull and placed in chilled low-calcium artificial CSF (ACSF) [in mM: 26 NaHCO_3_, 1.25 NaH_2_PO_4_, 10 glucose, 125 NaCl, 3 KCl, 5 MgCl_2_, and 1 CaCl_2_, pH 7.2–7.4 (290–310 mOsm)]. After chilling (5 min), the brain was trimmed to a block containing the hypothalamus and optic nerves. The brain was sliced in the coronal plane on a vibratome (Leica Microsystems) at a thickness of 300 μM. The slices were kept in aerated (95% O_2_/5% CO_2_) ACSF [in mM: 26 NaHCO_3_, 1.25 NaH_2_PO_4_, 10 glucose, 125 NaCl, 3 KCl, 2 MgCl_2_, 2 CaCl_2_, pH 7.2–7.4 (290–310 mOsm)] at room temperature (1.5 h) before being transferred to an electrophysiological recording chamber.

### Whole-cell patch-clamp electrophysiology

Slices were placed in a recording chamber (PH-1; Warner Instruments) attached to the stage of a fixed-stage upright differential interference contrast (DIC) microscope (Olympus). The slices were superfused continuously (2 ml/min) with ACSF aerated with 95% O_2_/5% CO_2_. The whole-cell patch-clamp recordings from the SCN were taken with recording electrodes. These micropipettes (typically 6–9 MΩ) were pulled from glass capillaries (World Precision Instruments) on a multistage puller (P-97; Sutter Instruments) and filled with the standard solution. The standard solution contained the following (in mM): 112.5 K-gluconate, 1 EGTA, 10 Hepes, 5 MgATP, 1 GTP, 0.1 leupeptin, 10 phosphocreatine, 4 NaCl, 17.5 KCl, 0.5 CaCl_2_, and 1 MgCl_2_. The pH was adjusted to 7.25–7.30, and the osmolality was adjusted between 290 and 300 mOsm. Recordings were obtained with the AXOPATCH 200B amplifier (Molecular Devices) and monitored online with pClamp (version 10; Molecular Devices). To minimize changes in offset potentials with changing solutions, the ground path used a KCl agar bridge. Each of the cells was determined to be within the SCN by directly visualizing the location of the cell with DIC microscopy. Cells were approached with slight positive pressure (2–3 cm H_2_O). The pipette was lowered to the vicinity of the membrane while maintaining positive pressure. After forming a high-resistance seal (2–10 GΩ) by applying negative pressure, a second pulse of negative pressure was used to break the membrane.

The access resistance of these cells ranged from 10 to 30 MΩ in the whole-cell voltage-clamp configuration, whereas the membrane capacitance was typically between 6 and 18 pF. Data were not collected if access resistance was >50 MΩ or if the value changed significantly (>20%) during the course of the experiment. In these studies, we used a 70% compensation using positive-feedback correction. The junction potentials between the pipette and the extracellular solution were canceled by the voltage offset of the amplifier before establishing a seal and were not further corrected. Series and input resistance were monitored repeatedly by checking the response to small pulses in a passive potential range. The standard extracellular solution used for all experiments was ACSF. Solution exchanges within the slice were achieved by a rapid gravity feed delivery system. Currents traces were recorded with pClamp using the whole-cell voltage-clamp recording configuration and then analyzed using Clampfit (version 10; Molecular Devices). I-V curve was examined using a voltage-step protocol in the whole-cell voltage-clamp configuration. The protocol consisted of the baseline at −70 mV, followed by progressively depolarized potentials (−120 to 30 mV, 10 mV steps). Current measurements were performed in control solution and after measuring spontaneous firing rate (SFR) in each cell. SFR were recorded with pClamp for 1 min using current clamp in the whole-cell patch configuration. After breakthrough of the membrane, data were obtained within 1 min. Membrane capacitance and resting membrane potential were also examined. No current was injected during recording. From 1 min recording, the membrane potential was measured in the intervals between the action potentials. Recordings were performed under room temperature. For SFR, under holding current (*I*=0), the voltage was monitored throughout the experiments. SFR were analyzed using the Clampfit and MiniAnalysis program (Synaptosoft). The software was used to automatically record the number and peak amplitude of action potentials recorded in the gap-free mode of the pClamp software. Each automatically detected event was then manually checked to ensure that the baseline and peak were accurately determined. The mean frequency and amplitude of action potentials were then calculated for each neuron.

### Real-time monitoring of bioluminescence

After 3-NP or vehicle treatment, wheel running activity of mice was evaluated as described above and the power of the rhythm noted. SCN (middle of the rostro-caudal axis) and hippocampus (anterior) were dissected from 300 μm coronal brain sections, along with heart atria and liver explants. Explants were transferred on to Millicell membranes (0.4 μm, PICMORG50, Millipore) resting on 1.2 ml of recording media that contained freshly added 0.1 mM luciferin (sodium salt monohydrate, Biosynth), and the 35 mm dishes were sealed using autoclaved high-vacuum grease (Dow Corning). Tissue explants were immediately inserted in the Lumicycle photometer (Actimetrics), and bioluminescence was monitored at 37°C for 7 consecutive uninterrupted days. Raw bioluminescence values were normalized by first subtracting the recorded baseline, then subtracting a running average of 24 h of this baseline-subtracted bioluminescence, and finally performing a 2-h smoothing average. Period, amplitude, and first calculated peak for phase relationships were calculated as previously described (Loh et al., 2011).

### Histology

Both the striatum and SCN of 3-NP (*n*=10) and vehicle (*n*=10) were examined. Mice were anesthetized by isoflurane (Clipper Distributing) and brains were dissected, fixed with 4% PFA at 4°C overnight, and cryoprotected in 20% sucrose in PBS (pH 7.4). Then, 20 μm cryostat (Thermo Fisher Scientific) coronal brain sections were made. Sections were washed for 5 min with PBS (pH 7.4, three times) and mounted on slides immediately. Sections were then dried overnight, stained with Cresyl Violet or hematoxylin and eosin staining, dehydrated with ascending concentrations of ethanol, and cover-slipped. We defined the striatum and SCN using stained mouse brains. For each mouse, images were captured from each of three regions (rostral, central and caudal striatum/SCN) using the AxioVision camera system (Carl Zeiss). Three tissue sections from each striatum and SCN (rostral, central, and caudal aspects of striatum/SCN) were chosen and images were taken. The representative sections were obtained from at least four animals. All positive cells within the striatum and SCN of these regions were counted manually at 40× with the aid of a grid (4×9). All positive cells within the grid were counted equally without regard to the intensity of the staining. Counts were done by two observers blind to treatment protocol and the results were averaged. Striatum ventricle and SCN size were also measured by AxioVision (Carl Zeiss).

### Statistical measurements

The datasets were analyzed by tests for equal variance and normal distribution to help select the appropriate test. Student's *t* test was used to compare two groups. The remainder of the datasets were analyzed by one-way ANOVA. If significant group differences were detected by the ANOVA, then a *post hoc* analysis was applied. Equal-variance test was performed for behavioral analysis. For all tests, values were considered significantly different if *P*<0.05. All tests were performed using SigmaPlot (Systat Software). Values are shown as means + or ± S.E.M.

## RESULTS

### Rhythms in wheel-running behavior are disrupted in some 3-NP-treated mice

**Figure 1:**
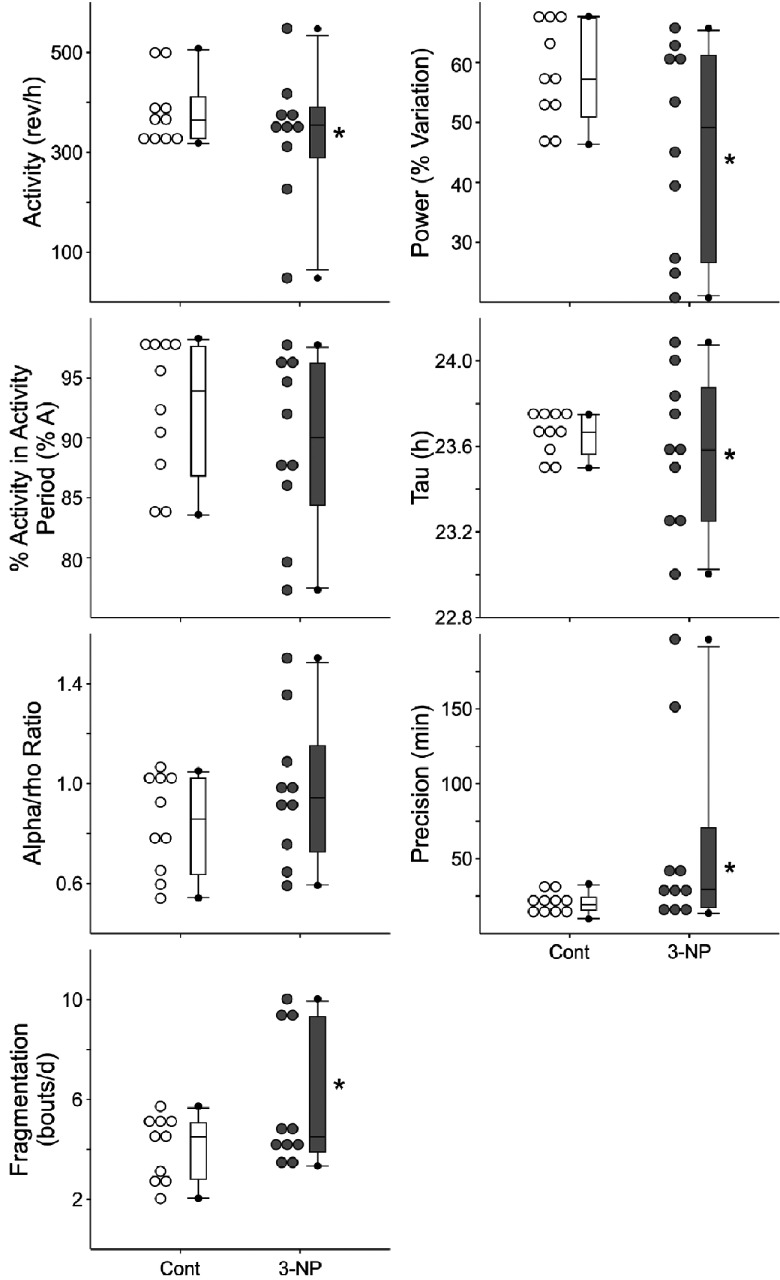
Diurnal and circadian rhythms of wheel-running behavior are disrupted in some 3-NP-injected mice Mice were placed individually in cages with wheel-running, and wheel-running activity was recorded under different lighting conditions. Dot density plot and box plot in wheel running activity under DD. Overall, we did not observe significant reductions in the amount of activity, power, precision, fragmentation or free-running period of the treated mice. On the other hand, there was a significant increase in the variability in these key circadian parameters in the 3-NP-treated mice. The increase in variability was mostly driven by three mice in the treated group which were profoundly impacted by the 3-NP treatment. *P<0.05, equal variance test vs controls.

**Figure 2:**
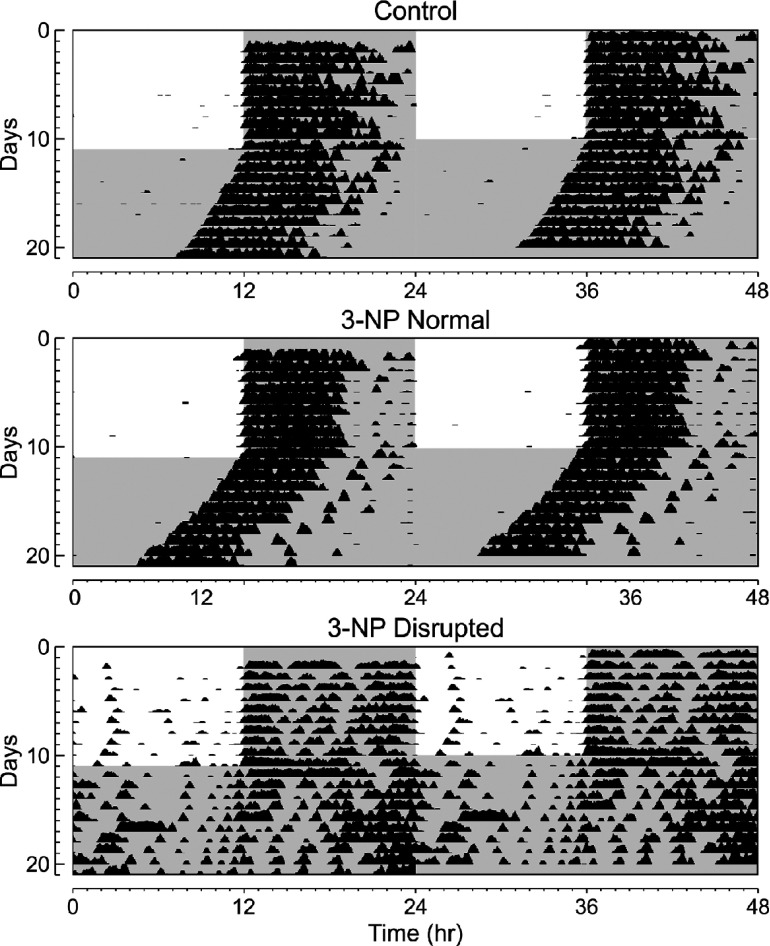
Examples of wheel-running behavior are shown Each horizontal row represents an activity recorded for a 24-h day. Successive days are plotted from top to bottom. Shaded panels represent the dark portion of the LD cycle. By conversion, the data is double plotted. Top panel: example of the wheel-running activity recorded from control mouse held in LD (12:12 h) and then released into DD. Middle panel: 3-NP-injected mice which showed normal activity. Bottom panel: 3-NP-injected mice which showed disrupted activity.

We used wheel-running activity to determine the impact of the mitochondrial complex II-specific toxin 3-NP on diurnal and circadian rhythms of behavior (Figure 1). In the first study, we compared locomotor activity rhythms in saline-injected control mice (*n*=10) and 3-NP-treated mice (*n*=10). Before the treatments, we confirmed that there were no differences in baseline activity rhythms between the groups (Supplementary Figure S1A at http://www.asnneuro.org/an/006/an006e133add.htm). Following 3-NP treatment (a daily dose of 15 mg/kg daily for a total of 5 days), we did not observe significant changes in the amount of activity, power, precision, fragmentation or free-running period of the treated mice (Supplementary Table S1 at http://www.asnneuro.org/an/006/an006e133add.htm). On the other hand, there was a significant increase in the variability in these key circadian parameters in the 3-NP-treated mice (Figure 1). The increase in variability was mostly driven by three mice in the treated group which were profoundly impacted by the 3-NP treatment. Using the periodogram analysis, this subset of treated mice (3/10) had a power (% variation) of less than 30. The 3-NP-injected mice exhibited a normal magnitude of light-induced phase shifts (Supplementary Figure S1). Overall, our data indicate that most 3-NP-treated mice exhibited essentially normal rhythms, while others exhibit a fragmented, low amplitude rhythm (Figure 2).

### Rhythms in sleep behavior are disrupted in some 3-NP-treated mice

**Figure 3:**
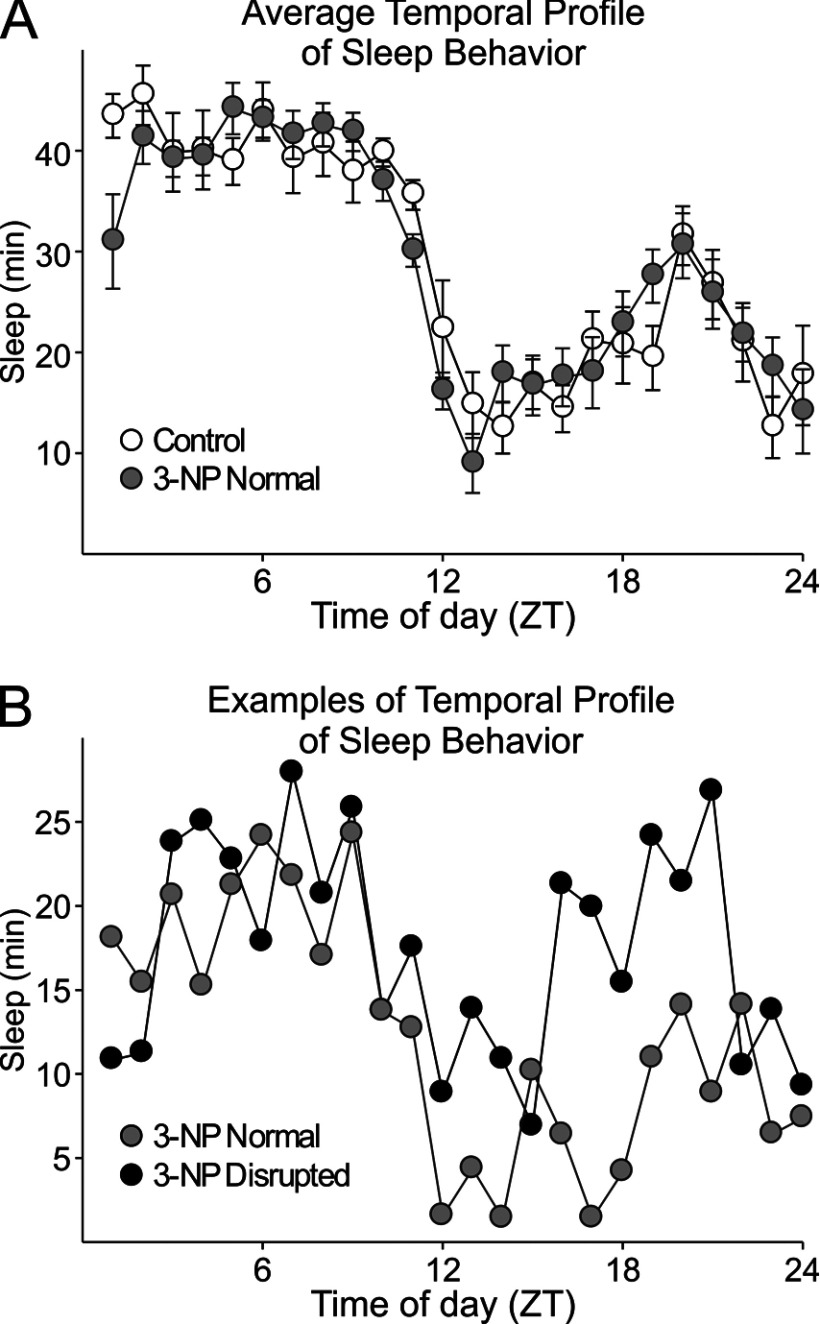
Rhythms in sleep behavior are disrupted in some 3-NP-treated mice Video recording in combination with an automated mouse tracking analysis software was used to measure sleep behavior. Top panel: average waveforms of the amount (min) of sleep per h between control (*n*=8) and 3-NP-treated (*n*=8) mice. Overall, we did not see significant differences in the temporal patterning of sleep. Some treated animals had difficulty initiating sleep. Bottom panel: provides an example of the sleep pattern recorded from a 3-NP-treated mouse with a disrupted sleep rhythm compared with a treated mouse with a normal pattern of sleep.

We used video recording to measure sleep as defined by time spent immobile, in combination with automated mouse tracking analysis software. Next, we compared behavioral sleep in control mice (*n*=8) and 3-NP-treated mice (*n*=8). Overall, the average waveforms of hourly immobility-defined sleep were broadly similar but did show evidence of difficulty initiating sleep (Figure 3, top panel). As nocturnal creatures, mice typically spend the majority of the daylight hours inactive. When examining individual animals, two of the eight 3-NP-treated mice lost the typical pattern of more sleep during the day (Figure 3, bottom panel).

### During the sleep phase, 3-NP-treated mice are more active

**Figure 4:**
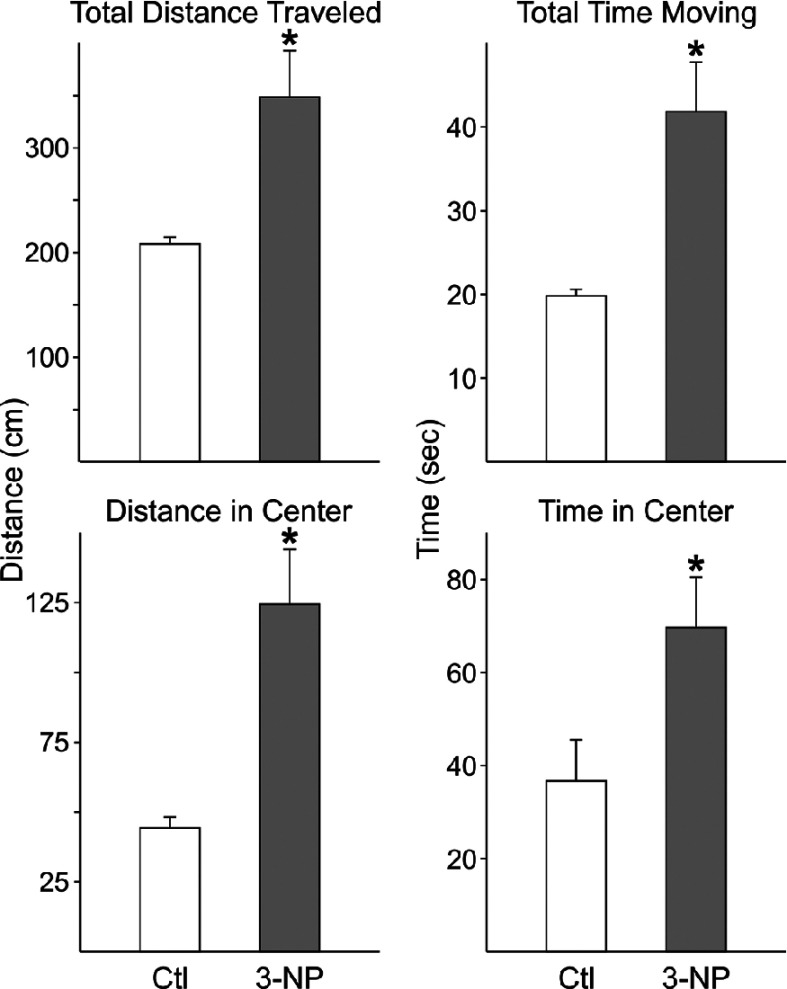
The 3-NP-treated mice show evidence for hyperactivity during sleep phase Open-field test was performed during the animals normal sleep time (ZT 2–6) in saline-injected control mice (*n*=7) and 3-NP-treated mice (*n*=7). * indicates *P*<0.05 as measured by a Mann–Whitney U test. There were no significant differences in other parameters measured such as vertical or stereotyped movements between groups.

The fragmentation of the activity/rest cycle suggests that at least some of the mice may have difficulty inhibiting movement during sleep. Therefore, we next compared locomotor activity in open field test during normal sleep time (ZT 2–6) in saline-injected control mice (*n*=7) and 3-NP-treated mice (*n*=7) (Figure 4). In this cohort, the 3-NP-treated mice exhibited a significant (*P*<0.05 as measured by the Mann–Whitney U test) increase in the total distance traveled (T=30; *P*=0.016), time spent moving (T=18, *P*=0.56), distance traveled in center of cage (T=15, *P*=0.008) and time spent in center of cage (T=15; *P*=0.008). There were no significant differences in other parameters measured such as vertical or stereotyped movements between groups. Therefore the treatment does appear to reduce sleep-associated inactivity.

### PER2-driven rhythms in bioluminescence were disrupted in some 3-NP-treated mice

**Figure 5:**
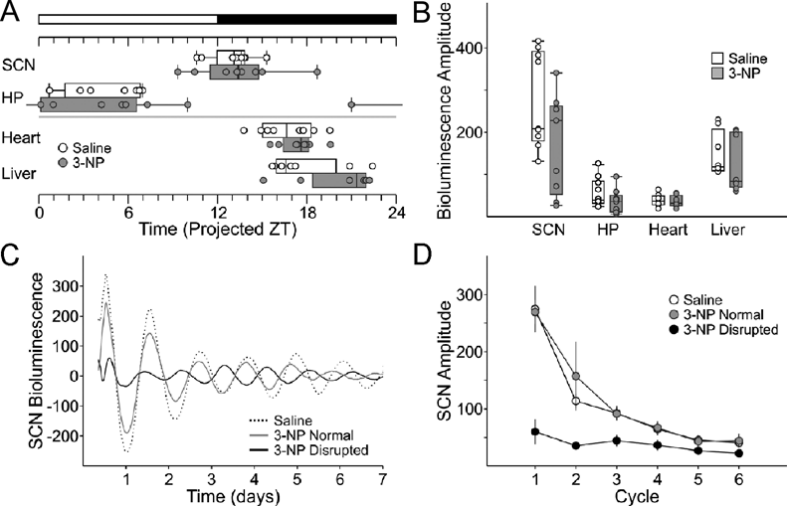
The amplitude of PER2::LUC bioluminescence rhythms was damped in a subset of 3-NP-injected mice (**A**) Phase relationship between the explants as determined by the first calculated peak of the rhythm in PER2::LUC bioluminescence. We see increases in variability in phase. (**B**) The amplitude of the rhythm in PER2::LUC bioluminescence. (**C**) Representative traces of PER2::LUC bioluminescence from the SCN of control (dashed), and two 3-NP-treated mice. (**D**) Four out of the 10 SCN explants from 3-NP-treated mice showed abnormally low amplitude of PER2::LUC bioluminescence that persisted for the duration of recording (black). The unaffected SCN explants (6/10; grey circles) had similar first and subsequent amplitudes of PER2::LUC bioluminescence to the explants from control (white).

The increase in variability in most circadian behavioral parameters in 3-NP-treated mice suggested that molecular clockwork responsible for the generation of circadian rhythms may be disrupted. To test this, we used a reporter mouse line that tracks PER2 expression using a fusion luciferase protein (PER2::LUC; Yoo et al., 2004) and measured the phase of central clock in the SCN and other oscillations known to be dependent on the SCN including the hippocampus, heart, and liver. The resulting data was similar to what we observed with the behavioral assay. Overall, we found that there were no significant differences in the amplitude (Figure 5A) or phase (Figure 5B) of the PER2::LUC bioluminescence rhythms that we recorded. The periods of the rhythms in bioluminescence measured from the SCN were different between treatment groups (control: 25.77±0.24, *n*=8; 3-NP: 24.83±0.24, *n*=8; *t* test, T=2.77, *P*=0.01). However, further examination of the individual SCN rhythms in bioluminescence revealed that a subset of the treated mice (*n*=4/8) had a clear damped rhythm in bioluminescence that persisted through the duration of the recording (Figures 5C and 5D). Again, these data suggest that while some 3-NP-treated mice exhibited essentially normal PER2 rhythms, others exhibit low-amplitude rhythms in the SCN.

### Excitability of SCN neurons was reduced in the behaviorally impacted 3-NP-injected mice

**Figure 6:**
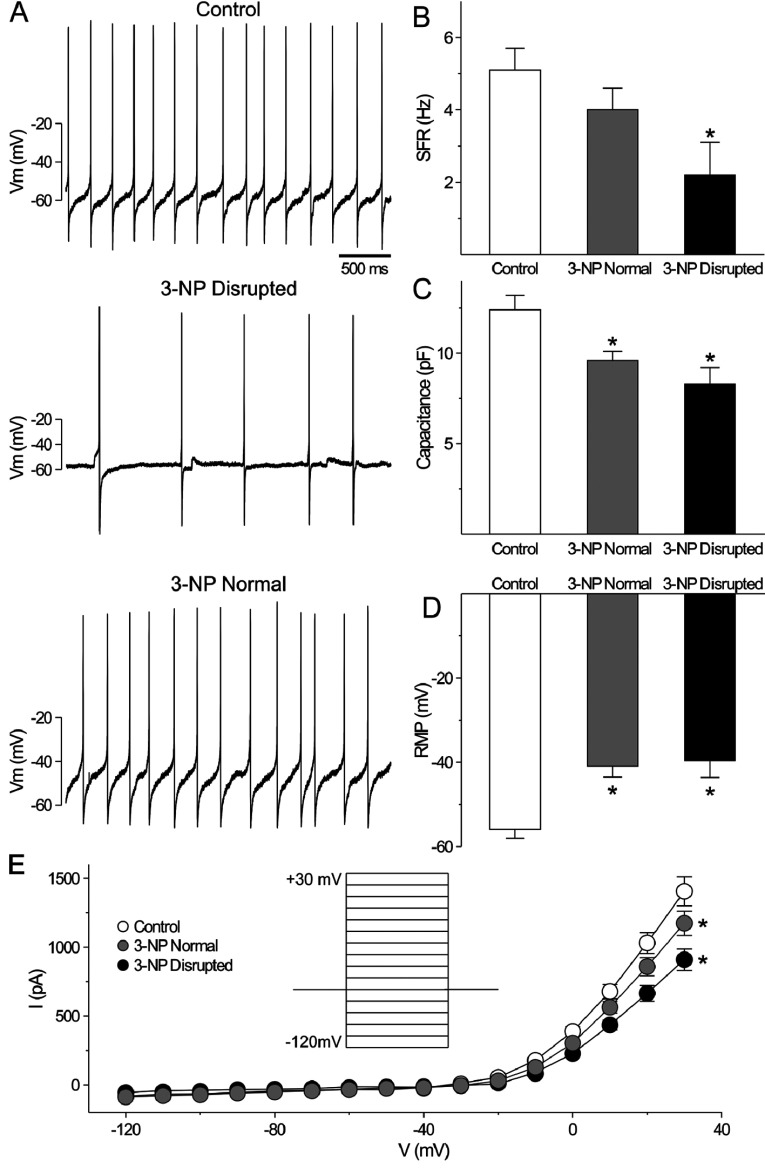
The daily peak of spontaneous firing rate in SCN neurons is reduced in some 3-NP-treated mice We first examined wheel running activity of a cohort of 3-NP-treated mice (*n*=9) and determined whether the circadian behavior was significantly reduced (power <30% using periodogram analysis; 3 out of 9 mice). Then, using the current-clamp recording technique in the whole-cell configuration, we measured the SFR in dorsal SCN neurons during the day (ZT 4–6). (**A**) Representative examples of firing rate recorded during the day from each of the groups. (**B**) Histograms show the average firing rate during the day for each group: control (white), 3-NP-treated with normal circadian behavior (grey), and 3-NP-treated with disrupted circadian behavior (black). Data are shown as means±S.E.M. *P<0.05 indicates significant difference analyzed by one-way ANOVA followed by Dunn's method (vs control). (**C**) Histograms show the average capacitance during the day for each group. (**D**) Histograms show the resting membrane potential (RMP) for each group. (**E**) Current–voltage relationship in the SCN neurons from mice of the three groups. Data are shown as means±S.E.M. *P<0.05 as measured by repeated measure ANOVA followed by Holm-Sidak method (vs. control).

SCN neurons are spontaneously active neurons with peak activity during the day. Given the variability in behavior, we first examined wheel running activity of a cohort of 3-NP-treated mice and determined whether the circadian behavior was significantly reduced (power<30% using periodogram analysis). Then using the current-clamp recording technique in the whole-cell configuration (Figure 6), we measured the SFR in dorsal SCN neurons in brain slices from control and 3-NP-injected mice: control (30 cells from 5 mice), 3-NP normal (40 cells from 6 mice), and 3-NP disrupted (12 cells from 3 mice). Recordings (1 min) were made during the day (ZT 4–6), and the resulting data were analyzed by one-way ANOVA. This analysis revealed a significant effect of treatment (H_2_=9.569, *P*<0.05). The SFR of mice judged behaviorally unaffected by 3-NP treatment (4.0±0.6 Hz) were indistinguishable from the wild-type mice (5.1±0.6 Hz), but SFR recorded from the behaviorally disrupted subset of 3-NP-treated mice was significantly lower than the control group (2.2±0.9 Hz, *post hoc* Dunn's Method, *P*<0.05, Figure 6). There was also a significant effect of treatment on the neuron's resting membrane potential [RMP; F_(2,68)_=12.404, *P*< 0.01]. *Post hoc* analysis revealed that there were no differences in the RMP between control (−41±3 mV) and behaviorally normal 3-NP-treated mice (−40±4 mV), whereas the RMP of SCN neurons from behaviorally disrupted mice was hyperpolarized (−56±2 mV; *post hoc* Holm-Sidak, *P*<0.05, Figure 6). Phase plot analysis confirmed that the RMP of the 3-NP group was higher than control (Supplementary Figure S2 at http://www.asnneuro.org/an/006/an006e133add.htm).

We examined the current–voltage relationship (*I*−*V*) from SCN neurons in the three groups. Two-way repeated measures ANOVA detected significant variation between groups [F_(2,1247)_=4.538, *P*< 0.01], voltages [F_(15,1247_)=237.922, *P*< 0.01), and interaction (group×voltage, F_(30, 1247)_=3.245, *p*< 0.01]. Measured at the peak (+30 mV), the currents in 3-NP normal and 3-NP disrupted were significantly lower than wild-type (*post hoc* Holm-Sidak, control vs 3-NP normal: *P*<0.05, control vs 3-NP disrupted: *P*<0.05, Figure 6). Therefore, the *I*−*V* curve was altered in the SCN neurons of the 3-NP-treated mice. Overall, the net impact of these physiological changes is that the day–night difference of SFR and RMP was lost in SCN neurons of 3-NP-treated mice that exhibit disrupted circadian behavior (Supplementary Figure S2).

### 3-NP treatment caused obvious pathology in striatum, but not the SCN

**Figure 7:**
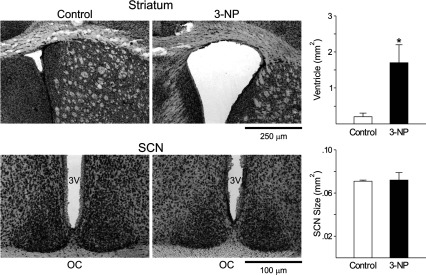
The volume of the striatal region is reduced by 3-NP injection To confirm that the 3-NP treatment was effective, we examined the striatum and SCN of a cohort of treated mice. Using Nissl and hematoxylin and eosin staining, we found that the striatum exhibited pathological damage in all of the treated mice (*n*=10/10). To provide one measure, the ventricle volumes adjacent to the striatum as well as the SCN were measured. Top left: photomicrographs (4×) of striatum Nissl staining sections. Top right: averaged ventricle size of striatum. *P<0.05, Student's *t* test (vs control). Bottom left: photomicrographs (10×) of SCN Nissl staining sections. 3V, 3rd ventricle, OC, optic chiasm. Bottom right: averaged SCN size. Data are shown at means+S.E.M.

Finally, to confirm that the 3-NP treatment was effective, we examined the striatum and SCN of a cohort of treated mice. Using Nissl and hematoxylin and eosin staining, we confirmed that the striatum exhibited pathological damage in all of the treated mice (*n*=10/10). The striatal area was significantly reduced in the 3-NP-treated group compared to controls (Student's *t* test: *P*<0.05, Figure 7). In contrast, the SCN region did not exhibit obvious cell loss as measured by either stain. We saw some evidence for damage in the ventricular region and it was difficult to rule out some more selective cell loss to the SCN cell population. Still these results demonstrate that the SCN does not exhibit gross pathology as a result of the 3-NP treatment and confirms that the treatment was successful in producing major damage to the striatum.

## DISCUSSION

Mitochondrial alterations are believed to play a critical role in the pathophysiology of neurodegenerative diseases. In the present study, we evaluated the circadian system of mice after inhibiting mitochondrial complex II of the respiratory chain with low doses of the toxin 3-NP. This protocol has been previously used by Dr Hernández to cause selective cell loss within the striatum (Rodríguez et al., 2010) and produces about a 20% reduction in complex II activity in the brain (E. Hernández-Echeagaray and Angélica Ruelas, unpublished work). By intent, this is a subtle treatment that we believe represents the type of insult that is more broadly experienced by people than the acute toxicity observed with higher doses of 3-NP (Liu et al., 1992; Ming, 1995). For a subset of the mice, the treatment resulted in severe deficits in circadian and sleep behavior along with hyperactivity during the normal sleep time. Similarly, as measured by PER2 driven bioluminescence, about half the mice exhibited a reduced amplitude oscillation when measured at the level of the central clock (SCN) and a change in free-running period that is normally a very sensitive indicator of disturbances in the molecular clockwork (Takahashi et al., 2008b). The reductions in the amplitude of circadian gene expression have recently been seen in patients with major depressive disorder (Li et al., 2013) and may be a negative indicator of cognitive function.

Given the variability in behavior, for the electrophysiological analysis, we first examined the wheel running behavior and separated the 3-NP-treated mice into those with essentially normal circadian behavior and those with a disrupted behavioral rhythm. Based on past experience, we used a power value from the periodogram analysis of less than 35% variance as an objective measure of a disrupted circadian rhythm. For comparison, the wheel running activity rhythms of young adult C57BL/6 mice have power values around 60. The mice with a disrupted circadian behavior exhibited a significant reduction in spontaneous electrical activity in SCN neurons during the daytime. These mice no longer exhibit the daily rhythm in neural activity which is the hallmark feature of SCN neurons and is critical for the central clock to communicate with the rest of the organisms (Schwartz et al., 1987; Yamaguchi et al., 2003; Colwell, 2011). This reduction in daytime activity is likely to underlie the disrupted behavioral rhythms in these mice. We have seen a reduction in daytime electrical activity in the SCN of aging mice (Nakamura et al., 2011) as well as in mouse models of HD and PD (Kudo et al., 2011a, 2011b). In each of these cases, the strength of the behavioral rhythms was significantly reduced in parallel with the reduction in behavior. One of the striking aspects of our data set that we cannot explain is the variability in the behavioral response. In several cohorts of treated mice, we kept finding around 20% of the treated mice exhibited significant circadian deficits. A subset of mice also exhibited SCN pathophysiology that we assume is responsible for the behavioral disruption. The treated mice are all C57BL/6 from the same colony and were essentially genetically identical. The 3-NP was administered i.p. once a day for 5 days and, while it is hard to rule out variability in dosing, we feel that this is an unlikely explanation for the variability. But we do not know why some mice are more vulnerable than others and are left to speculate about epigenetic contributions to the response. C57BL/6 mice are also known to exhibit substantial resistance and variability in response to quinolinic acid treatment (e.g. McLin et al., 2006; Strong et al., 2012). Prior work has shown SCN neurons to be particularly resistant to excitotoxic damage (Colwell and Levine, 1996; Bottum et al., 2010). In a comparison that is particularly relevant to the present study, the same excitotoxic treatment that cause necrosis and death of striatal neurons had no acute impact on SCN neurons (Colwell and Levine, 1996). In this context, it is worth noting that the 3-NP treatment caused significant striatal damage in every mouse that we examined (*n*=10/10). This finding suggests that we can disassociate the circadian deficits from the striatal damage. There has been a growing interest in understanding the role of the basal ganglia circuit in the control of sleep-wake behavior (e.g. Lazarus et al., 2012). At least one previous study found that bilateral lesions made in the striatum results in fragmentation of the sleep/wake cycle (Qiu et al., 2010). While not the focus of the present study, our results indicate that it is possible to express robust sleep/wake rhythms even with fairly extensive striatal damage.

This work raises the possibility that mitochondrial dysfunction can contribute to the sleep/circadian dysfunction seen so commonly in neurodegenerative diseases. Sleep disruptions are common in patients with neurodegenerative diseases and occur early in the disease progression (Schlosser et al., 2012; Morton, 2013; Willison et al., 2013). Many of these patients exhibit problems in the timing of sleep including difficulty sleeping at night and stay awake during the day which is indicative of an underlying circadian dysfunction. In genetic models of these diseases, circadian dysfunction has been confirmed (Morton et al., 2005; Gonzales and Yin, 2010, Bedrosian et al., 2011; Kudo et al., 2011a, 2011b; Oakeshott et al., 2011; Roh et al., 2012; Kantor et al., 2013; Fisher et al., 2013; Loh et al., 2013). A common feature of both aging and neurodegenerative disease is evidence for associated deficits in mitochondrial function (Beal, 2005; Cookson, 2012; Nakamura et al., 2012; Youle and van der Bliek, 2012; Subramaniam and Chesselet, 2013).

It seems quite possible that mitochondrial dysfunction caused by 3-NP treatment could underlie the pathophysiology observed in the present study. The 3-NP treatment decreases complex II-dependent mitochondrial respiration which would decrease mitochondrial efficiency and thereby increase reactive oxygen species byproducts. One of the distinguishing features of SCN neurons is the ability to generate action potentials during the day when nocturnal organisms are relying on energy reserves. This spontaneous activity imposes a significant mitochondrial burden especially in the subset of SCN cells that are secretory and have broad action potentials and significant calcium entry. During the day, the SCN are one of the brains ‘hot spots’ with high levels of 2-deoxyglucose consumption (e.g. Schwartz and Gainer, 1977). The argument has been made that the selective vulnerability of some cell populations to diseases such as PD may be the unfortunate combination of spontaneous activity coupled with low calcium-binding capabilities (Surmeier and Schumacker, 2013). In contrast, SCN neurons are loaded with calcium-buffering proteins such as calbindin (e.g. Kriegsfeld et al., 2008) which may prevent the cell death in this region. Still the oxidative stress caused by the mitochondrial toxin could well explain the pathophysiology. Recent work suggests that the spontaneous electrical activity of SCN neurons is very sensitive to changes in the redox state (Wang et al., 2012). In addition, it is worth noting that K^+^ channels are sensitive to oxidative damage (Sesti et al., 2010; Cotella et al., 2012). Increased daytime firing rates are dependent on K^+^ channel currents, and therefore oxidative damage to the channels, which are mediating these currents in the SCN, may underlie the reduced daytime firing rates observed in the 3-NP model. Therefore, the most promising explanation for decreased SCN firing frequency in the 3-NP model is decreased mitochondrial function and increased oxidative stress. As far as we know, this is the first demonstration that chronic treatment with this mitochondrial toxin alters excitability in the SCN or other brain regions although previous work has shown that 3-NP treatment decreases excitatory synaptic transmission in the striatum (Rossi et al., 2006). Future examination of these targets provides new hypotheses to explore the mechanism underlying reduced SCN firing frequency and in turn may provide new insights into disease progression and disease prevention.

## Online data

Supplementary data
