# Supplementary material for: Circadian dysfunction in response to in vivo treatment with the mitochondrial toxin 3-nitropropionic acid
Source: ASN Neuro. 2014 Jan 13;6(1):e00133. doi: 10.1042/AN20130042 (PMC3891360; doi:10.1042/AN20130042)
Supplement: Supplementary data [file an006e133add.pdf]

ASN NEURO RESEARCH ARTICLE

ASN NEURO 6(1):art:e00133.doi:10.1042/AN20130042

OPEN ACCESS

Circadian dysfunction in response to *in vivo* treatment with the mitochondrial toxin 3-nitropropionic acid

Takashi Kudo*[1], Dawn H. Loh*, Yu Tahara*†, Danny Truong*, Elizabeth Hernández-Echeagaray‡ and Christopher S Colwell*

*Laboratory of Circadian and Sleep Medicine, Department of Psychiatry and Biobehavioral Sciences, University of California, Los Angeles, CA, U.S.A.

†Department of Physiology and Pharmacology, School of Advanced Science and Engineering, Waseda University, Tokyo 162–8480, Japan

‡Neurofisiología del desarrollo y la neurodegeneración, Unidad de Biomedicina, Universidad Nacional Autónoma de México

SUPPLEMENTARY DATA

Table S1 **Detailed analysis of activity after the injection**

rev, revolutions.

Analysis	Control ($n = 10$)	3-NP ($n = 10$)
LD 12/12 h		
Activity (rev/h)	378 ± 28	357 ± 37
Power (% variation)	60.6 ± 3.0	59.0 ± 3.9
Activity in night (% A)	97.7 ± 0.4	93.9 ± 1.0
Activity onset (ZT)	12.09 ± 0.04	11.96 ± 0.18
Activity offset (ZT)	23.23 ± 0.14	21.87 ± 0.33
α/ρ ratio	0.87 ± 0.02	0.80 ± 0.03
Precision (min)	14 ± 3	18 ± 9
Fragmentation (bouts/day)	3.4 ± 0.4	4.2 ± 0.5
DD		
Activity (rev/h)	380 ± 21	334 ± 41
Power (% variation)	57.9 ± 2.6	46.0 ± 5.4
% Activity in activity period (% A)	92.5 ± 1.8	89.5 ± 2.3
Tau (h)	23.66 ± 0.03	23.58 ± 0.11
α/ρ ratio	0.84 ± 0.06	0.97 ± 0.09
Precision (min)	20 ± 2	56 ± 20
Fragmentation (bouts/day)	4.1 ± 0.4	5.8 ± 0.8

[1] To whom correspondence should be addressed (email tkudo@mednet.ucla.edu).

© 2014 The Author(s) This is an Open Access article distributed under the terms of the Creative Commons Attribution Licence (CC-BY) (http://creativecommons.org/licenses/by/3.0/) which permits unrestricted use, distribution and reproduction in any medium, provided the original work is properly cited.

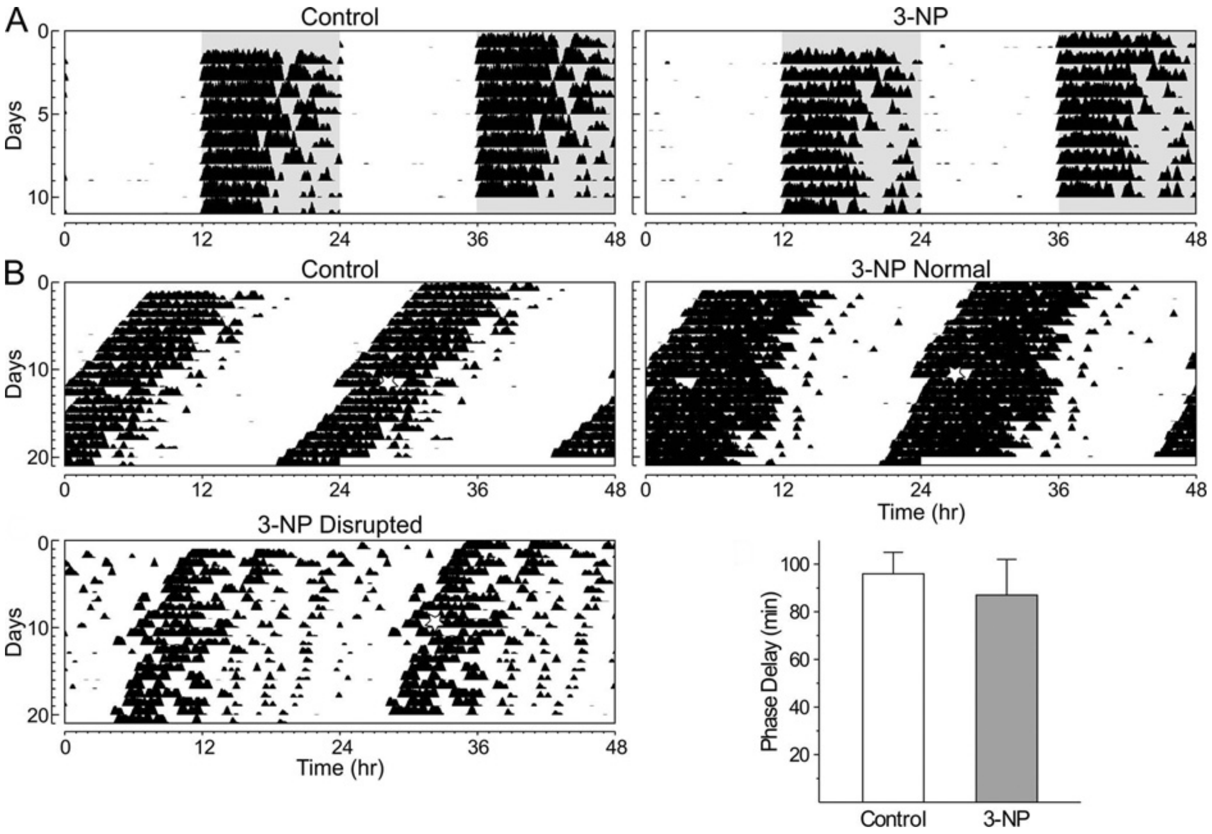

Figure S1 **Additional wheel-running activity data**
(**A**) There are no differences of circadian rhythms of wheel-running behavior before the injections. The examples of the wheel-running activity recorded from control mouse held in LD (12:12 h) before vehicle or 3-NP treatment. (**B**) Examples of light-induced phase shifts for control, 3-NP normal, and 3-NP disrupted groups in DD. Mice in DD were exposed to light (100 lux, 10 min duration) at CT 16 (indicated by symbol) and the resulting phase delays were measured. Bottom right: averaged light-induced phase shift. Data are shown at means + S.E.M.

© 2014 The Author(s) This is an Open Access article distributed under the terms of the Creative Commons Attribution Licence (CC-BY) (http://creativecommons.org/licenses/by/3.0/) which permits unrestricted use, distribution and reproduction in any medium, provided the original work is properly cited.

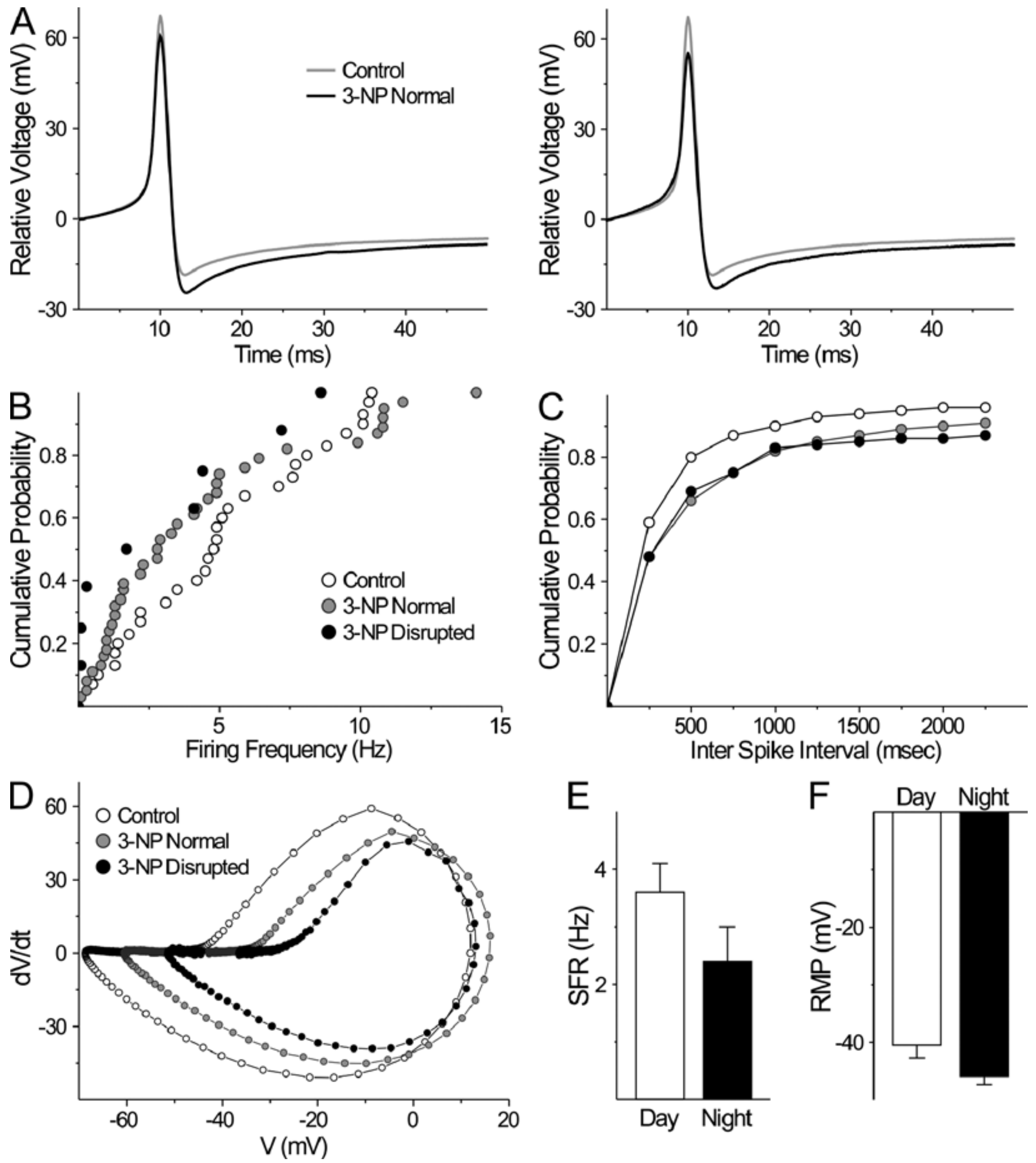

Figure S2 **Additional electrophysiological data**
Using the current-clamp recording technique in the whole-cell configuration, we measured the SFR in dorsal SCN neurons during the day and night. (**A**) Representative examples illustrating the averaged action potentials. (**B** and **C**) Cumulative probability plot shows a decrease of firing frequency in 3-NP-injected mice (**B**) and an increase of inter spike interval of 3-NP-injected mice (**C**). (**D**) Phase plot analysis which shows the 3-NP impact on the action potential wave form. (**E**) Histograms plotting average firing rate during the day and night in 3-NP-injected mice. (**F**) Histograms plotting averaged RMP recorded during the day and night in 3-NP-injected mice.

© 2014 The Author(s) This is an Open Access article distributed under the terms of the Creative Commons Attribution Licence (CC-BY) (http://creativecommons.org/licenses/by/3.0/) which permits unrestricted use, distribution and reproduction in any medium, provided the original work is properly cited.
